# Early career perspectives of young Dutch cardiologists

**DOI:** 10.1007/s12471-021-01561-y

**Published:** 2021-04-19

**Authors:** V. M. M. Vorselaars, A. C. van der Heijden, R. Joustra, W. R. Berger, G. P. J. van Hout, G. F. L. Kapel, R-J. Nuis, P. Woudstra, S. R. D. Piers

**Affiliations:** 1 Department of Cardiology, St. Antonius Hospital, Nieuwegein, The Netherlands; 2 Department of Cardiology, Leiden University Medical Centre, Leiden, The Netherlands; 3 Department of Cardiology, Radboud University Medical Centre, Nijmegen, The Netherlands; 4 Department of Cardiology, Onze Lieve Vrouwe Gasthuis, Amsterdam, The Netherlands; 5 Department of Cardiology, University Medical Centre Utrecht, Utrecht, The Netherlands; 6 Department of Cardiology, Medisch Spectrum Twente, Enschede, The Netherlands; 7 Department of Cardiology, Erasmus Medical Centre, Rotterdam, The Netherlands; 8 Department of Cardiology, Amsterdam University Medical Centre, location AMC, Amsterdam, The Netherlands

**Keywords:** Career choice, Employment, Contract

## Abstract

**Background:**

There are nationwide concerns about the unemployment rate among young Dutch cardiologists and the increase in temporary positions. Therefore, the aim of this study was to investigate the unemployment rate in this subgroup as well as the length of time between the end of their training and the acquisition of a permanent position.

**Methods:**

All cardiologists who completed their training between January 2015 and December 2018 were invited to fill in an online questionnaire about their demographic characteristics, professional profile and employment status. The unemployment rate was calculated and Kaplan-Meier curves were used to determine the time between the end of training and the first permanent contract.

**Results:**

In total, 174 participants were included (mean age 35 ± 3 years, 64% male, median follow-up 2.3 years (interquartile range 1.4–3.2 years)). The unemployment rate was 0.6% (*n* = 1). Only 12 participants (7%) started their career with a permanent position. The percentage of cardiologists with a temporary position was 82%, 61% and 33% at 1, 2 and 3 years, respectively. The percentage of cardiologists with a temporary position did not differ with regard to age, gender, holding a PhD degree or type of teaching institution attended (academic vs non-academic). Forty-four per cent of participants perceived the current job market to be problematic.

**Conclusions:**

The unemployment rate among young cardiologists in the Netherlands was low between 2015 and 2018. The vast majority of cardiologists start their career on a temporary contract. Three years later, 33% still hold temporary positions. Due to the resultant job insecurity, many young cardiologists describe the job market as problematic.

## What’s new?

- The unemployment rate among young cardiologists in the Netherlands was low between 2015 and 2018.
- The majority of cardiologists start their career on a temporary contract.
- Three years after starting their career as cardiologists, 33% still hold temporary positions.
- Many young cardiologists describe the job market as problematic.

## Introduction

Since 1999 the Dutch government has controlled the number of medical specialists by regulating the inflow into medical specialty training. Due to the anticipated increase in the demand for healthcare, the annual number of cardiologists in training has risen sharply [1]. Although the inflow of new cardiologists in training has stabilised in recent years, a mismatch with the outflow of retiring cardiologists remains. As a consequence, the number of cardiologists almost doubled from 654 in 2000 to 1266 in 2020. While this increase in the total number of active cardiologists has in part been essential to meet the growing demand for cardiovascular care, it has also led to a surplus of young cardiologists on the current labour market. Therefore, there is nationwide concern about unemployment among young cardiologists [1].

In 2016 the Dutch Federation of Young Medical Specialists (*De Jonge Specialist) *wrote a report on the short-term career perspectives of young Dutch medical specialists. In addition to an unemployment rate of 2.1%, an increase in temporary contracts was observed, with over 50% of contracts being temporary [2]. In 2017 Vis et al. observed a low unemployment rate (1.6%) among 189 young cardiologists. Most cardiologists started with a temporary position; however, after 4 years 93% had a permanent contract. Predictors of permanent employment within 1 year were male gender and being trained in an academic teaching hospital [3]. More recently, in 2019, the number of job vacancies per 100 cardiologists was among the lowest of all medical specialties [4].

To monitor the current trends in employment among young cardiologists, we as the board of the Junior Association of the Netherlands Society of Cardiology (*De Juniorkamer*) initiated a new survey. The primary objectives were to investigate the unemployment rate and the time required to find permanent employment. Secondary objectives were to compare career perspectives with regard to gender, professional profile and type of teaching institution attended.

## Methods

### Study population and survey

A digital questionnaire was sent to all cardiologists who completed their training between January 2015 and December 2018. The data were collected anonymously and participants were asked to provide written informed consent. The questionnaire included questions on demographic characteristics, professional profile (teaching institution, area of expertise, PhD degree, completion of a fellowship), current employment status (employed vs unemployed, permanent vs temporary position) and previous jobs. Participants were also asked whether they were experiencing any problems with the current job market for young cardiologists and whether, with hindsight, they felt there were gaps in the cardiology training programme.

If participants did not respond or written informed consent was not obtained, data were considered missing for the primary outcome. Additionally, current work status of the non-responding participants was obtained via representatives of their teaching institution.

### Statistical analysis

All statistical analyses were performed using IBM SPSS Statistics version 25 for Windows (versions 25.0, SPSS Inc., Chicago, IL, USA). Descriptive statistics were used to describe participants’ characteristics and type of contract. Categorical variables were expressed as number (percentage) and continuous variables as mean ± standard deviation (SD) or median (interquartile range, IQR). Kaplan-Meier survival analyses were used to assess the time required to obtain a permanent position. Furthermore, cumulative incidences were calculated and groups were compared using the log-rank statistic. A Cox regression analysis was used to analyse the association between time to permanent position and age, gender, holding a PhD degree and type of teaching hospital attended. A *p*-value of < 0.05 was considered statistically significant.

## Results

### Demographic data

Between 1 January 2015 and 31 December 2018, 223 residents completed their training programme. Questionnaires were sent to all 223 cardiologists (134 (60%) trained in an academic teaching hospital and 89 (40%) in a non-academic teaching hospital). In total, 174 (79%) participants gave written informed consent and were included for further analyses. Median follow-up was 2.3 years (IQR: 1.4–3.2 years). Mean age was 35 ± 3 years, and 64% were male. Furthermore, 63% were trained at an academic teaching institution and 55% had a PhD degree. The baseline characteristics are shown in Table 1.

**Table 1:** Baseline characteristics of young cardiologists

Baseline characteristics	*n* = 174
Age (years)	35 ± 3
Male	112 (64%)
*Teaching hospital*	
Academic	110 (63%)
Non-academic	64 (37%)
PhD degree	(55%)
*Area of expertise*	
General cardiology	18 (10%)
Imaging	51 (29%)
Electrophysiology	17 (10%)
Interventional cardiology	38 (22%)
Heart failure	13 (8%)
Devices	23 (13%)
Adult congenital heart disease	5 (3%)
Intensive care	4 (2%)
Other	5 (3%)
*Fellowship*^a^	100 (58%)

Continuous variables are presented as mean ± SD and categorical variables as *n* (%)

^a^Cardiologists who are either currently enrolled in or have already completed a fellowship

### Work status

**Fig. 1:**
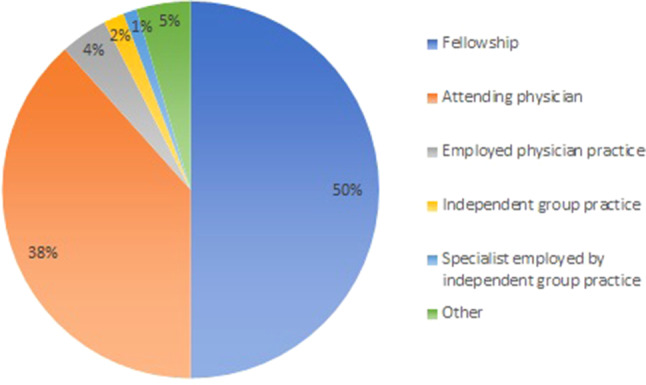
First job of young cardiologists

Only one participant reported being unemployed, corresponding to an unemployment rate of 0.6%. A total of 91 participants (52%) had a temporary position and 82 (47%) a permanent contract. As demonstrated in Fig. 1, for the majority of subjects the first working experience as a cardiologist was in a temporary position (88%), either as a fellow (50%) or attending physician (38%). Only 12 participants (7%) started with a permanent position, either at an independent group practice (*n* = 3, 25%), an employed physician practice (*n* = 7, 58%) or as a specialist employed by an independent group practice (*n* = 2, 17%). The remaining 5% (*n* = 7) were classified as ‘other’, consisting of research positions, practicing abroad or employed in an advisory position.

**Fig. 2:**
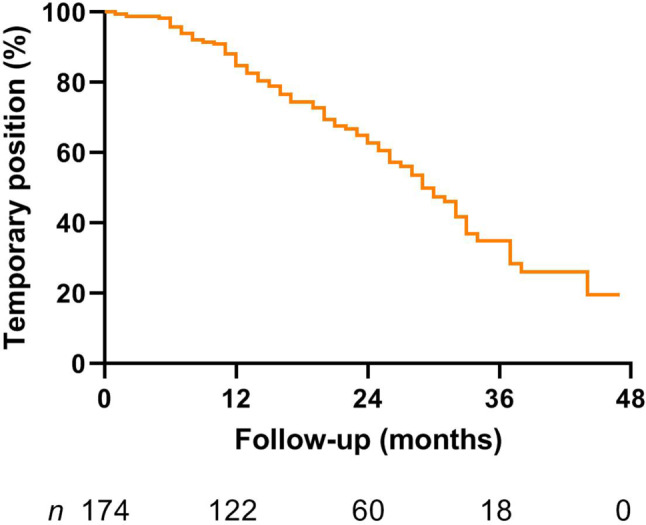
Temporary positions amongst young cardiologists

One year after completion of their training, 82% (95% CI 76–88%) of participants had a temporary position. After 3 years of follow-up this number had declined to 33% (95% CI 23–43%), as illustrated in Fig. 2. When all cardiologists who were either enrolled in or had completed a fellowship were excluded (*n* = 100), the percentage of cardiologists with a temporary position was 73% (95% CI 63–83) at 1 year after completion of their cardiology training and 20% (95% CI 5–34) at 3 years after their training.

Of 32 non-responding participants, employment data were retrieved from representatives of the teaching hospitals. None of these cardiologists were unemployed; after including them in the total cohort the total unemployment rate was 0.5%. The type of employment could be established in 27 of these cardiologists. When these data were added to the total cohort, 102 (51%) cardiologists had a temporary and 98 (49%) a permanent position.

### Profile and work status

**Fig. 3:**
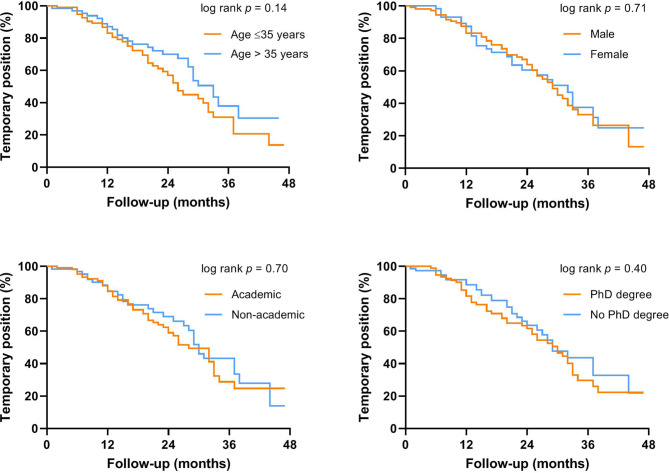
The percentage of temporary positions by demographic and professional profile features of young cardiologists

Table 2 summarises the subgroup analyses of employment status in young cardiologists. After 3 years of working experience the incidence of temporary employment did not differ with regard to age, gender, type of teaching hospital or PhD degree (Fig. 3).

**Table 2:** Factors associated with the percentage of temporary positions amongst young cardiologists after 3 years of working experience

	Temporary positions			
	3 years (95% CI)^a^	*p*-value	HR (95% CI)^b^	*p*-value
*Age*				
≤ 35 years	30% (17–43)	0.14	Reference 1.41 (0.95–1.87)	0.14
> 35 years	35% (18–52)			
*Gender*				
Male	37% (21–54)	0.71	Reference 1.09 (0.64–1.54)	0.72
Female	31% (18–44)			
*Academic teaching hospital*				
Yes	28% (14–41)	0.70	Reference 1.09 (0.64–1.54)	0.70
No	42% (26–57)			
*PhD degree*				
Yes	27% (14–40)	0.40	Reference 1.21 (0.76–1.64)	0.41
No	42% (27–57)			

*CI* confidence interval, *HR* hazard ratio

^a^Based on cumulative incidence as calculated by Kaplan-Meier survival analyses

^b^Calculated using Cox regression analyses

In a subgroup analysis of cardiologists aged above 35 years old, males and females were as likely to have a permanent position after follow-up (HR: 0.75 (95% CI 0.01–1.49), *p* = 0.44). When young cardiologists were stratified according to both PhD degree and type of teaching hospital, the percentage of temporary positions was lowest in cardiologists with a PhD degree who were trained at an academic teaching hospital (24%) compared to the other groups (38–44%), but the difference was not statistically significant (*p* = 0.82).

### Personal opinion of young cardiologists

Forty-four per cent of participants perceived the current job market to be problematic. In the subgroup of cardiologists with a permanent position, 27% reported problems. The preferred mean working time was 0.89 ± 0.11 full-time equivalent (FTE), which was similar to the mean reported working time (0.91 ± 0.12 FTE).

The greatest concern of young cardiologists is the shortage of permanent positions, causing young cardiologists to switch jobs frequently. In line with this concern, the presence of multiple attending physicians in one hospital without the possibility for permanent employment was perceived to be problematic. The above-mentioned concerns are described both by cardiologists with temporary and permanent positions. Most cardiologists describe research, management and leadership as important gaps in their training programme.

## Discussion

The primary objective of this study was to re-evaluate current career perspectives of young cardiologists in the Netherlands. The main results are: (1) there was a very low rate of unemployment between 2015 and 2018 (0.6%, *n* = 1); (2) the vast majority of young cardiologists start their career as a fellow or attending physician with a relatively high rate (33%) of temporary contracts after 3 years of follow-up; (3) hallmark demographic and career characteristics such as age, gender, PhD degree or type of teaching hospital were not found to have a significant influence on the time required to obtain a permanent position.

When compared to the previously published analysis of young cardiologists in the Netherlands, the unemployment rate tends to be slightly lower (0.6% vs 1.6%), while the percentage of temporary contracts at the start of a career is higher (88% vs 77%). The percentage of temporary contracts after 3 years of follow-up is comparable (33% vs 30–31%).

These data indicate that, although there is hardly any unemployment, the percentage of temporary contracts among starting cardiologists has gradually increased over the last few years and the time between completing training and a first permanent contract is long. In contrast to previous findings, we did not observe gender or teaching hospital to have an influence on the time required to obtain a permanent position.

Multiple aspects could have a significant influence on the high incidence of temporary employment. Firstly, subspecialisation is becoming increasingly important in cardiology. The percentage of cardiologists taking fellowships could therefore increase over time. This is in line with the high percentage of cardiologists with fellowships as a first contract both in the current analysis and previously published results (50% vs 46%). However, the percentage of attending physicians as a first contract increased sharply when compared to previous analyses (38% vs 24%). Therefore, increased subspecialisation only partly explains these results.

Secondly, in parallel with the increase in the demand for cardiovascular healthcare, the number of trained cardiologists has increased strongly over the last few years [1]. At the same time, a nationwide reorganisation of the financial structure within hospital care has taken place and many reorganisations and hospital mergers have occurred. Furthermore, financial cuts in hospital healthcare costs and the prevention of growth in healthcare expenses by the government have led to an uncertain future for Dutch hospitals [4]. It could be hypothesised that those financial factors, as well as the increased number of available cardiologists (‘supply/demand mismatch’), may explain the high number of temporary positions.

As mentioned, the previous analysis by Vis et al. revealed an effect of gender as well as of teaching hospital with regard to the time to acquire a permanent position [3]. In the current study we could not replicate these findings. This may possibly be due to the emancipation of women in the field of cardiology and an increasing general acceptance for men to work part-time, although this interpretation remains speculative. The lack of influence of the type of teaching hospital may suggest a uniform method of education or an improved balance in demand for cardiologists trained in academic and non-academic institutions. However, given the non-significant difference of 14%, it could also be that larger group numbers would indeed reveal a difference.

In the present study we did not analyse the association between fellowships and career perspectives due to the limited follow-up (median 2.3 years) and because most fellowships are coupled with temporary employment contracts, making it difficult to perform an unbiased analysis. Another factor complicating such analyses is the definition of fellowships, which varies greatly between hospitals and subspecialisations. It is not uncommon for temporary positions described as attending physician in the past to be currently referred to as fellowships. This could have biased the current analyses. Uniform description of fellowships for several subspecialties in cardiology may provide clarity on this matter. With this goal in mind, a fellowship working group was recently founded by the Netherlands Society of Cardiology (NVVC).

The number of young cardiologists starting their career as a specialist permanently employed by an independent group practice was found to be low. It is expected that this number will rise as young cardiologists gain more working experience. To adequately assess the incidence of specialists permanently employed by an independent group practice further follow-up is needed.

A significant number of young cardiologists perceive the current job market as problematic. This is mainly due to the financial and geographical insecurity that comes with temporary job positions at a time in life that often involves multiple structural changes in a short timeframe (e.g. marriage, family planning). The lack of true unemployment suggests that there is a strong public need for cardiologists to meet the high demand of cardiovascular care. Therefore, from the perspective of an aging community requiring cardiovascular care, it is of great importance to look after the working climate of our cardiologists.

Job insecurity is not unique to cardiology. As reported in 2016 by the Dutch Federation of Young Specialists (*De Jonge Specialist*), there is unemployment (2.1%) and an increase in temporary contracts (over 50% temporary contracts) in many other medical specialties [2]. However, more recent data on the career perspectives of young medical specialists need to be collected. Moreover, job uncertainty is also not unique to the current younger generations outside the field of medicine (generation X and Y). Young professionals were found to have higher levels of job insecurity and overall career concerns compared to their older counterparts [5]. However, in contrast to many non-medical professions, cardiologists (and many other medical specialists) are trained for more than a decade for a highly specialised profession, without many other job options. This is not only a time-consuming but also an expensive training programme, which thus emphasises the need for a balance between the inflow of young cardiologists and the demand in the labour market.

Temporary employment comes with less autonomy at the workplace, decreased control over the work environment and possibly less contact with and support from colleagues. These factors induce stress, potentially leading to decreased job satisfaction and even burnout [6]. Unfortunately, for physicians less job satisfaction can lead to medical errors. For healthcare organisations, high physician job satisfaction is eventually also of economic value, as it reduces absence due to illness and prevents early retirement from medical practice [7]. The observed high rate of temporary contracts is therefore worrying and highly undesirable.

The ongoing COVID-19 pandemic may potentially lead to a decline in the number of cardiac patients and hence fewer financial healthcare problems. For young cardiologists this may result in more uncertainty concerning the labour market and fewer available permanent positions.

### Future perspectives

In order to improve the career perspectives of young cardiologists we believe that it is essential to regulate and match the inflow of (specialised) cardiologists to the actual demands of the labour market. To achieve this goal the following data and measures are vital:

1. Complete and live data on the number of active cardiologists (number of FTE) and job vacancies. The committee *commissie behoefte beraming* (Committee for the Estimation of Staffing Needs) has recently been assigned to create a national data acquisition system that will provide these live data.
2. Accurate estimations of the annual outflow of cardiologists (due to retirement).
3. Define and make fellowships uniform on a national basis, which is the primary goal of a working group recently initiated by the NVVC.
4. Align the number of fellowships with the job vacancies for subspecialists (e.g. interventional cardiologists).

In addition, an advisory dashboard, started by the NVVC or the Federation of Medical Specialists (FMS), could help young cardiologists with job application training or in planning their career.

### Limitations

Several limitations should be addressed. First, the questionnaire was short to ensure a high response rate. Unfortunately, 21% still did not respond despite several requests, which could have resulted in a selection bias. However, in 65% of non-responders we managed to acquire additional key information, which showed no differences in unemployment rate and the proportion of temporary contracts. Second, our follow-up period is too short to compare the effect of fellowships on the probability of permanent employment for these young cardiologists.

### Conclusions

The unemployment rate among young cardiologists in the Netherlands was low between 2015 and 2018. The vast majority of cardiologists start their career on a temporary contract. Three years after starting their career as a cardiologist, 33% still hold temporary positions. Due to the resulting job insecurity, many young cardiologists describe the job market as problematic.
